# Self-Regulated Writing Strategy Use When Revising Upon Automated, Peer, and Teacher Feedback in an Online English as a Foreign Language Writing Course

**DOI:** 10.3389/fpsyg.2022.873170

**Published:** 2022-04-19

**Authors:** Lili Tian, Qisheng Liu, Xingxing Zhang

**Affiliations:** ^1^School of Foreign Languages, Renmin University of China, Beijing, China; ^2^Handan Vocational College of Science and Technology, Handan, China

**Keywords:** self-regulated learning, writing strategy, peer feedback, automated feedback, teacher feedback, L2 writing, revision

## Abstract

Research investigating the intricacies of how self-regulated writing strategies are used in a finely focused area of the second language (L2) writing process is still lacking. This study takes a mixed-methods approach to explore Chinese English as a Foreign Language (EFL) learners’ use of self-regulated writing strategies when revising based on automated, peer, and teacher feedback in an online EFL writing context. Thirty-six Chinese university learners filled in three questionnaires (one per feedback source). In addition, four learners followed a think-aloud protocol while revising and responding to a stimulated recall interview to provide further data. The results revealed that learners employed an array of self-regulated writing strategies to attain their feedback revision goals. Learners used more cognitive strategies when revising based on automated feedback compared with peer and teacher feedback and more motivational strategies when revising based on teacher feedback. The think-aloud data and stimulated recall interviews coincided with the quantitative findings. Textual analysis revealed that feedback type and quantity were associated with self-regulated writing strategy use.

## Introduction

Self-regulated learning (SRL) has been a constant focus on the second language (L2) research since its inception (Zimmerman, 1986, 2000; Dörnyei, 2005; Oxford, 2011). Under SRL, learners actively regulate their affect, cognition, and behavior in goal-oriented learning (Zimmerman, 2013). SRL is a multidimensional construct that entails an array of interrelated cognitive, emotional, motivational, and contextual factors (Teng and Zhang, 2021). SRL strategies are deployed to regulate cognition, metacognition, social behavior, and motivation to achieve learning goals. Researchers have confirmed the merits of SRL in L2 writing by exploring their relationship with writing performance (Csizér and Tankó, 2017; Bai and Wang, 2021), individual differences (Teng, 2021), and the effectiveness of strategic intervention (Chamot and Harris, 2019; Guo et al., 2021). However, most studies in this area have made broad assumptions based on large-scale questionnaires. Therefore, “the intricacies of how learners make use of (SRL) strategies in more finely focused areas of the learning process” is still under-researched (Rose, 2015, p. 322).

Feedback has received an increasing amount of attention among L2 writing researchers because of its effectiveness in improving writing performance (e.g., Hyland and Hyland, 2006; Li and Vuono, 2019). Research on learner engagement with feedback and feedback uptake has enhanced our understanding of feedback and subsequent revision processes (Zhang and Hyland, 2018). English as a Foreign Language (EFL) learners’ active engagement in feedback and revision processes has affective, cognitive, and behavioral dimensions (Tian and Zhou, 2020). However, to date, few studies have focused on learners’ SRL strategy use in this finely focused area of L2 writing (Andrade and Evans, 2013; Xiao and Yang, 2019). Current research on writing strategy use has investigated feedback handling and revising strategies as one section of the overall writing process and has overlooked the multidimensional nature of feedback and revision processes themselves. Feedback revision tasks are highly self-regulated and autonomous in nature, requiring learners to plan, monitor, and evaluate their own feedback revision process (Zhang and Qin, 2018), especially in online EFL writing contexts (Jansen et al., 2017). As Xiao and Yang (2019, p. 41) claimed, “Feedback and self-regulation are two intricately interrelated aspects of a broader learning process.” Accordingly, a deep dive into learners’ strategy use during the feedback uptake decision-making process would provide refined insights into how learners regulate their cognitive, affective, and behavioral engagement with feedback.

To address this research gap, this study takes a mixed-methods approach to explore the self-regulated writing strategy use of Chinese EFL learners when they are conducting revisions in response to automated, peer, and teacher feedback in an online EFL writing course.

## Literature Review

### Self-Regulated Learning and Learning Strategies in L2 Writing

The SRL, initially proposed by Zimmerman (1986) as metacognitively, motivationally, and behaviorally active participation by learners in their own learning process, has since been defined as “the ways that learners systematically activate and sustain their cognitions, motivations, behaviors, and affects, toward the attainment of their goals” (Schunk and Greene, 2018, p. 1). This multidimensional and goal-oriented concept emphasizes learners’ active participation in regulating their own learning and the systematic and dynamic interaction of cognitive, metacognitive, motivational, affective, and environmental factors in this process (Teng and Zhang, 2021).

The process-oriented and goal-directed nature of L2 writing, supported by cognitive writing models (Flower and Hayes, 1981), highlights the relevance of the SRL perspective to the writing process. This approach recognizes that self-regulated writing processes involve the integration of cognitive, metacognitive, affective, and environmental factors (Panadero, 2017). Large-scale questionnaire studies have shown the value of SRL strategy use in relation to L2 writing performance (Teng and Zhang, 2016; Csizér and Tankó, 2017). Such studies have revealed the individual differences that correlate with SRL strategy use, such as personality (Jackson and Park, 2020), self-efficacy (Teng, 2021), motivational belief (Bai and Wang, 2021), and mindset (Bai and Wang, 2020). SRL strategy intervention studies have further revealed the effectiveness of explicit cognitive and metacognitive strategy use on L2 writing achievements (Hu and Gao, 2017; Chamot and Harris, 2019; Teng and Zhang, 2020). The longitudinal development of self-regulated writing strategies was also examined in mixed-methods research by Sasaki et al. (2018). They investigated the interaction of self-regulated writing strategies (e.g., global planning, local planning, and L1–L2 translation) with cognitive, affective, and environmental factors in L2 writing. Nonetheless, learners’ SRL strategy use in finely focused areas of the L2 writing process remains under-researched (Andrade and Evans, 2013; Xiao and Yang, 2019).

### Self-Regulated Learning Strategies and Feedback Revision in L2 Writing

Based on a paradigm shift from “what writers write” to “how writers write,” process-oriented writing (Zamel, 1976) views writing as a cyclical process based on rounds of revisions driven by feedback from peers or teachers, which aims to improve text quality (Kroll, 2001). During revision in response to feedback, learners actively participate in decision-making processes by employing several strategies and meta-strategies for text improvement. For example, the study of Yu and Lee (2016) reveals that Chinese EFL learners have employed five major strategies while engaging in peer feedback activities (using L1, employing L2 writing criteria, adopting rules of group activity, seeking help from teachers, and playing different roles) to facilitate their peer interaction and successful revision learning. Writers’ active participation and dynamic use of strategies associated with feedback and revision to regulate their own writing are compatible with the concept of SRL, which emphasizes how learners effectively control their own learning process: “To develop SRL, learners must be actively engaged in the decision-making processes related to revision and editing” (Andrade and Evans, 2013, p. 50). Hattie and Timperley (2007, p. 94), in their model using feedback to enhance learning, proposed that self-regulation “can lead to seeking, accepting, and accommodating feedback information.” Studies have shown that self-regulated learners can interpret and evaluate feedback from different sources to facilitate their own learning goals (Lam, 2015).

The SRL strategies learners deploying to handle feedback from multiple sources are essential for developing self-regulated writers. Studies have revealed a significant correlation between feedback handling and revising strategies during the various stages of feedback revision and writing competence (Bai, 2015; Teng and Zhang, 2016; Bai and Wang, 2020). For example, Teng and Zhang (2016, p. 681) developed and validated the four categories of writing strategies for an SRL questionnaire in the Chinese EFL writing context, namely, cognitive strategies (e.g., text processing and course memory strategies) refer to skills students use to process the information or knowledge in completing a task; metacognitive strategies (e.g., idea planning and goal-oriented monitoring and evaluation) refer to the skills used to control and regulate learners’ own cognition and the cognitive resources they can apply to meet the demands of particular tasks; social behavior strategies (e.g., feedback handling and peer learning) involve individuals’ attempts to control their learning behavior under the influence of contextual and environmental aspects; motivational strategies (e.g., motivational self-talk, interest enhancement, and emotional control) refer to the procedure or thoughts that students apply purposefully to sustain or increase their willingness to engage in a task. Feedback handling, open to peer or teacher feedback and improving students’ own writings accordingly, is one of the six self-regulated writing strategies that significantly predicts EFL writing proficiency. Bai’s (2015) study on writing strategy intervention used an experimental research design and found that three strategies, i.e., text-generating, feedback handling, and revising, significantly contribute to the improvement of writing competence after intervention. Feedback handling and revising strategies were embedded in the whole writing process of the writing strategy-based instruction lessons, from setting writing goals to revising the essay and getting and responding to feedback. Subsequently, Bai (2018) examined self-regulated writing strategies among 32 Singapore primary pupils based on the think-aloud protocol and found that revising strategies were frequently used, such as revising mechanics, revising ideas at clause/sentence level, revising during drafting, revising after drafting, and revising by deletion and substitution.

However, most of these studies have examined self-regulated writing strategies throughout the writing process (Flower and Hayes, 1981), incorporating planning, organizing, and monitoring in addition to revising (De Silva and Graham, 2015; Hwang and Lee, 2017; Sun and Wang, 2020). Revising strategies, in these studies, are categorized merely as cognitive strategies about how to revise (e.g., *revising mechanics*; *making lexical, morphological, and syntactic changes*; *revising ideas at the clause/sentence level*) rather than strategies and meta-strategies for making revising decisions based on feedback from external sources. Feedback handling strategies are simple statements aimed at feedback and revision activities in general (e.g., *I am open to teacher feedback on my writing*; *I read peers’ feedback*) rather than contextualized and fine-grained strategies used in response to feedback from different sources.

The notion of learner engagement with feedback considers both feedback characteristics and feedback uptake and facilitates our comprehension of feedback revision processes (Han, 2017; Suzuki et al., 2019). Consensus has been reached regarding learners’ multidimensional engagement with multiple feedback sources from cognitive, affective, and behavioral perspectives (Zhang and Hyland, 2018). Case studies that closely examined learner engagement with feedback have reported the use of cognitive and metacognitive strategies (Han, 2017; Tian and Zhou, 2020). When revising based on feedback, learners’ cognitive strategies utilize, e.g., linguistic, rhetorical, and discourse knowledge to revise a text with reference to writing lectures attended. Metacognitive strategies refer to specific idea-generating behaviors before revising, which aim at directing and monitoring revision activities. When engaging in feedback revision activities, learners might also need to ask for help from others and constantly regulate their motivation and maintain a positive frame of mind to complete the feedback revision tasks. Considering the multidimensional nature of learner engagement with feedback and revision (Zhang and Hyland, 2018), revising based on feedback likely entails multidimensional SRL strategy use (Teng and Zhang, 2021).

Furthermore, evidence from distance language learning supports the role of autonomy (Murphy, 2005). This implies that learners in online writing contexts where multiple forms of feedback are conveyed to learners through online platforms might need to devote more effort to planning, reflecting, monitoring, and evaluating during feedback revision processes. Previous studies in online contexts have examined either student and teacher perceptions of online writing courses having adopted electronic feedback (Tai et al., 2015; Litterio, 2018), or learner engagement with different feedback sources (Tian and Zhou, 2020). Those studies (e.g., Jansen et al., 2017) examining SRL in online contexts have mainly advocated the importance of SRL in L2 online learning, consisting of metacognitive skills, environmental structuring, help-seeking, time management, and persistence. Few studies have examined SRL strategy use in the feedback revision stage of the L2 writing process in an online writing context.

This study aims to fill this gap by examining SRL strategy use when revising based on automated, peer, and teacher feedback in an online EFL writing context through a mixed-methods approach. It intends to understand fine-grained strategy use and the driving force behind the feedback uptake decision-making process. The following research questions were examined.

RQ1: What self-regulated writing strategies are used by Chinese EFL learners when revising based on automated, peer, and teacher feedback in an online English writing context?
RQ2: How do learners deploy these self-regulated writing strategies?

## Materials and Methods

### The Online Writing Context

College English Writing II is an online English writing course at one of the top five universities in Beijing, China. The course is one of the three online writing courses in the target university and is an optional course for undergraduates. This course, run every spring term, aims to foster learners’ abilities in expository and argumentative writing. Learners are supposed to watch 16 online video lectures (40 min each), attend three face-to-face lectures, write three essay assignments, and complete three feedback revision tasks for each essay assignment. Learners who successfully complete all tasks gain course credits.

The three-stage feedback revision framework was specifically designed for this online English writing course to help learners improve their writing through multiple revisions. “Three-stage” refers to three different feedback sources, namely, an automated writing evaluation (AWE) program, peers, and the teacher. Each assignment topic undergoes the three-stage feedback revision procedure before a final essay is submitted. Learners can receive multiple feedback successively from the three sources and revise their essay drafts accordingly. AWE feedback is given by Pigai^1^, a widely used online AWE program in China (Bai and Wang, 2018; Tian and Zhou, 2020). Peer feedback pairs are randomly allocated by the online writing program, and teacher feedback is also conveyed online.

For this study, the second of the three essay assignment topics, an argumentative essay topic, was chosen as the target topic. By that time, learners were familiar with the feedback revision procedure and could devote themselves to the task. The assignment was as follows:

*Read the following statement about cultures: “The modern idea that all cultures are worthy of equal respect is illogical. Why should we give the same respect to cultures who have barbaric practices as those who are civilized?” Write an essay arguing for or against this statement.*

### Participants

A convenience sample of 52 first-year students enrolled in the online English writing course was invited to participate in the study. All participants were non-English majors from a wide range of subjects, such as philosophy, international relations, journalism, finance, business, and French. However, only 36 of them voluntarily completed all the three questionnaires at three different feedback-revision stages.

In addition to complete the questionnaires, four of the 36 participants (Table 1) voluntarily agreed upon invitation to follow think-aloud protocols and respond to stimulated recall interviews. Three of these four students were women, and they came from various subject backgrounds. An English Placement Test, administered to all first-year students upon entering the university, was available for use as a reference for their general proficiency level.

**TABLE 1:** Demographic information for the four case participants.

Pseudonym	Gender	Major	English placement test scores (maximum 100)
Zeng	Female	Philosophy	70
Li	Male	Business	57
Luan	Female	Finance	76
Wu	Female	Marxist Theory	59

### Instruments

#### Questionnaire

We adopted context-based and task-specific questionnaires to assess learners’ reported use of self-regulated writing strategies when revising based on AWE, peer, and teacher feedback. The questionnaires were contextualized in the online EFL writing course and administered for each of the three feedback sources. The questionnaire (see Appendix A) was mainly adapted from the Writing Strategies for Self-Regulated Learning Questionnaire (WSSRLQ) by Teng and Zhang (2016). The WSSRLQ is also aimed at Chinese EFL writers and entails four streams of strategies, i.e., cognitive, metacognitive, motivational, and social behavior strategies (Table 2), which embody the multidimensional nature of learner engagement with feedback.

**TABLE 2:** Questionnaire about self-regulated writing strategy when revising based on feedback.

Strategies	Denotation	Subcategories	Example items
Cognitive	Learner’s use of linguistic, rhetorical, and discourse knowledge to revise a text with reference to online and offline writing lectures	Text processing	*I check for grammar mistakes.*
		Course memory	*I revise according to words and expressions taught in online writing lectures*
Metacognitive	Specific pre-revision idea-generating behavior based on an arsenal of strategies to direct, monitor, or evaluate revising activities	Idea planning	*I evaluate the content to make revising decisions*
		Goal-oriented monitoring and evaluating	*I set up goals for myself to direct my feedback revision activities*
Social	Help from others in the revising environment	None	*I discuss with others to have more ideas to revise*
Motivational	Motivation to complete tasks and efforts to reduce distraction and emotion in the revising environment	Motivation	*I try to improve my English writing based on feedback I receive*
		Emotional control	*When I am feeling bored revising, I force myself to pay attention*

While adapting the WSSRLQ, we maintained Teng and Zhang’s (2016) cognitive and metacognitive strategy subcategories but revised the motivational and social strategy subcategories according to this study’s feedback revision context. Previous research findings were considered when adapting the questionnaire. We kept relevant items from Teng and Zhang (2016), revised items with reference to peer learning, emotional control, and revising strategies (Bai et al., 2014; Bai, 2015; Jansen et al., 2017), and inserted items related to the online learning context (Jansen et al., 2017). The internal consistency reliability of the questionnaire subscales was verified, with a Cronbach’s alpha value above the 0.70 threshold (*a* = 0.76; DeVellis, 2012). The questionnaire was revised iteratively following pilot trials before being administered to the 36 participants at the end of each feedback revision stage.

#### Think-Aloud Protocol

We adopted think-aloud protocols to assess learners’ spontaneous use of SRL strategies (Vandevelde et al., 2015; Bai, 2018). A think-aloud protocol, as “a recording of what is revealed in the participants’ voluntary reporting of what they are going through while they are engaged with a particular real problem-solving or learning task,” generates “truthful representations of participants’ mental activity or processes” (Zhang and Zhang, 2020, p. 303). The selected four participants received 15-min individual training sessions in advance. The authors first explained to the participants the aim and procedure of the protocol and then set examples before they independently practiced thinking aloud on an unrelated task. The training was continued until the participants expressed confidence in the process. All think-aloud protocol sessions were audio-recorded and individually performed by the participants in Chinese during each feedback revision stage.

#### Stimulated Recall Interview

We conducted stimulated recall interviews with the four selected participants after they completed all the feedback revision stages to clarify learners’ feedback revision decision-making. This enabled us to triangulate the interviews with the questionnaire and think-aloud protocol reports; triangulation “aims to collect multiple perspectives on an event so that the more complete understanding of the topic under examination can be gained” (Paltridge, 2020, p. 29). The interview guideline covers the following questions: “*Do you think peer feedback helps?*”; “*During think-aloud, you mentioned that you revised synonyms according to feedback from Pigai, but actually you only incorporated one example of synonym feedback from Pigai. Can you tell me why?*”; “*Why did you add 16 new sentences in this revised version?*”

### Data Collection

**FIGURE 1:**
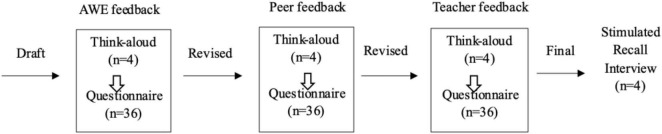
Data collection procedure.

We adopted a mixed-methods approach to investigate which self-regulated writing strategies are used and how they are used when learners revise in response to multiple feedback sources from AWE, peers, and the teacher over a month. Both retrospective and introspective data were collected (Figure 1) to capture learners’ spontaneous and reported writing strategy use during the three-stage feedback revision process. In each feedback revision cycle, the four selected participants followed the think-aloud protocol. At the end of each feedback revision cycle, all 36 learners completed the questionnaire. After all feedback revision stages were completed, the four selected learners responded to a stimulated recall interview. Therefore, in total, the data comprised 108 questionnaires (36 × 3), 4 stimulated recall interview transcripts, 12 sets of think-aloud data (4 × 3), 12 rounds of feedback from the 3 feedback sources (4 × 3), and 24 drafts and submitted essays from the 4 participants during the 3 feedback revision stages (4 × 3 × 2).

### Data Analysis

Data analysis involved quantitative analysis of questionnaires, content analysis of think-aloud and stimulated recall interview data, and textual analysis of 12 rounds of feedback and 24 essays. Questionnaire data were statistically analyzed using SPSS. The mean frequency of each of the four strategy categories was calculated for each case. Based on Kolmogorov–Smirnov (K–S) tests of normality (Table 3), parametric tests, such as repeated measures ANOVA, were conducted to examine strategy use differences across feedback sources.

**TABLE 3:** Kolmogorov–Smirnov tests of normality.

Strategies	AWE		Peer		Teacher	
			
	K–S (df)	Sig.	K–S (df)	Sig.	K–S (df)	Sig.
Cognitive	0.074 (36)	0.200	0.138 (36)	0.081	0.107 (36)	0.200
Metacognitive	0.129 (36)	0.138	0.089 (36)	0.200	0.077 (36)	0.200
Social	0.139 (36)	0.075	0.128 (36)	0.141	0.136 (36)	0.089
Motivational	0.98 (36)	0.200	0.117 (36)	0.200	0.134 (36)	0.100

After being transcribed verbatim by the third author, the think-aloud data were analyzed qualitatively with reference to self-regulated writing strategies. First, all authors conducted open coding to identify strategy categories and then matched them with the strategy category list in the questionnaire. A combination of bottom-up and top–down data mining methods was used to precisely identify feedback revision strategies in all stages, together with iterative identification. All disagreements were resolved among the authors based on the repeated reading of transcripts and rounds of discussions. Stimulated recall interview data were transcribed verbatim before being analyzed qualitatively to triangulate with the self-regulated writing strategies reported in the questionnaire and think-aloud data.

Textual analysis of feedback and revisions was based on a coding scheme (Table 4) adapted from Faigley and Witt (1981) and Allen and Mills (2016); the scheme was also adopted by Tian and Zhou (2020), who used it to calculate feedback and feedback uptake rates. In this approach, feedback that did and did not suggest changes in meaning was classified as meaning- and surface-level feedback, respectively. Feedback from AWE, peers, and teachers was coded accordingly. Revisions were first conducted by comparing draft and submitted essays at each of the three feedback revision cycles and stages. Subsequently, revisions were compared with feedback at each stage to differentiate feedback according to whether it resulted in revisions. Incorporated feedback was counted as feedback uptake; uptake rate was then calculated by dividing total feedback by feedback uptake.

**TABLE 4:** Feedback revision coding scheme and examples.

Feedback categories	Subcategories	Denotation	Examples
Surface-level feedback	Formal changes	Grammatical and mechanic feedback	*Original: Therefore, We should have*… *Feedback: “We” should not be capitalized*
	Meaning-preserving	Feedback that does not involve meaning changes at lexical, sentence, or paragraph levels	*Original: Cultural equality is a question of one’s choice Feedback: “question” can be replaced by synonyms such as problem or matter*
Meaning-level feedback	Meaning-related	Feedback involving meaning changes at lexical, sentence, or paragraph levels	*Original: Therefore, we should have the same respect to different cultures, no matter barbaric or civilized* *Feedback: What do the terms “barbaric” and civilized” mean? Explain them to readers*

## Results

### RQ1: What Are the Self-Regulated Writing Strategies Used by Chinese English as a Foreign Language Learners When Revising Based on Automated Writing Evaluation, Peer, and Teacher Feedback?

Thirty-six learners completed three questionnaires pertinent to the three feedback sources, retrospectively reporting their strategy use when revising. Table 5 shows that all learners employed all four strategies across the three feedback revision stages to a large extent. Frequencies ranged from 3.51 (social strategy based on peer feedback) to 4.44 (motivational strategy based on teacher feedback) along a 5-point Likert-type scale ranging from 1 (*not at all true of me*) to 5 (*very true of me*).

**TABLE 5:** Average frequency of strategy use according to the three feedback sources.

Strategies	AWE	Peer	Teacher

	Mean (*SD*)	Mean (*SD*)	Mean (*SD*)
Cognitive	4.16 (0.51)	3.97 (0.53)	3.99 (0.598)
Metacognitive	3.83 (0.50)	3.78 (0.61)	3.91 (0.64)
Social	3.66 (0.66)	3.51 (0.79)	3.66 (0.96)
Motivational	4.11 (0.59)	3.86 (0.77)	4.44 (0.51)

Qualitative analysis of both think-aloud and stimulated recall interview data showed that the four analyzed cases, regardless of their proficiency levels, employed all strategies to regulate their feedback revision activity. This is in accordance with the quantitative findings on strategy use and actual revision practice.

#### Cognitive Strategy Use

Wu, a low-proficiency student who employed cognitive strategies when revising based on peer feedback, is a good case in point. She checked the original draft to evaluate the validity of the feedback and revised it afterward. For example, she stated as follows in the think-aloud data:

He [the peer] said that this sentence in the fourth paragraph reads a bit Chinglish [original sentence: “Although there is some truth to this concern, it may not all be true, as Mill has done, by…, we can …”]. I look at the sentence and check whether it is inappropriate. I have adopted his feedback and revised the sentence [revised sentence: “I think it may not be true. As Mill has done, by…, we can …”]. The sentence now looks more natural.

She employed a series of cognitive strategies when revising based on peer feedback. She checked grammatical mistakes, cohesiveness among sentences, and clarity of expression of the content and then tried to use different English sentence patterns. In the revised version, she corrected the grammatical mistake by separating the original sentence into two and shortened the first part of the sentence by deleting the Chinglish part.

Another example is Zeng, a high-proficiency student, who reported the use of cognitive strategies following teacher feedback in her interview: “I think we should read through teacher feedback many times before revising. This is what I did; I also read the online lecture PowerPoint files and the handouts the teacher gave in offline lectures.” It is clear that Zeng referred to what she learned in both online and offline lectures to ensure her revision was effective.

#### Metacognitive Strategy Use

Zeng, a high-proficiency student, received no written corrective feedback from her peer but an overall positive comment. However, she employed a series of metacognitive strategies to self-regulate her peer feedback revision activities. Think-aloud data show that she reflected in detail about each aspect of the positive comment, evaluated it in correspondence with her essay, categorized its different aspects, analyzed the reasons for the strengths listed in the comment, and made decisions on continuing to use the identified writing skills in future.

I now start to look at his comment—well-written, very coherent, attractive opening paragraph, and sufficient support from well-selected evidence. First is coherence … Next is my effective opening paragraph. This is what I deliberately focus on. I used a comparison in my opening this time, and it works. I think I will continue to use this method.

Low-proficiency students also reported using metacognitive strategies. Li, e.g., established goals when revising based on teacher feedback. She contrasted the to-be-revised essay with her previous essay to better locate the problem. In the think-aloud data, she stated as follows:

The teacher gave me a lot of comments for this essay. From these comments, I can tell there are plenty of problems. I summarize the reasons by comparing this essay with my previous one and know that the biggest problem of this essay is expression … this essay is a piece of argumentation in which we need to express our views and thoughts.

She also monitored her revision process by progressively tackling each of the teacher’s comments. Another example is Li, who reported her constant use of the Internet or e-dictionary resources when revising based on automated feedback.

#### Social Strategy Use

Luan, the most proficient of the four cases, reported the use of social strategies to help with decision-making when revising based on teacher feedback. When she came across teacher feedback that she was unsure about, she turned to a native speaker, who helped her figure out how to approach revising the text and interpreting the feedback.

The next feedback relates to when I wrote “we are outsiders observing it through color blind glasses.” Here the teacher said, “this is informal language.” I am a bit puzzled. I then asked a native speaker who told me that “tinted glasses” is what I want to express. What I wrote is different from what I wanted to express. Actually, when we didn’t use English phrases in authentic ways, we didn’t know their meaning in English.

#### Motivational Strategy Use

The four cases reported in their interviews that they tried to improve their English writing based on the feedback they received. They also exhibited motivational and emotional control when revising based on teacher feedback in the think-aloud data. Zeng (high proficiency) said in the interview that she pushed herself further when she began to wander or lose interest when revising. She explained that teacher feedback is demanding. Sometimes, she disagreed with her teacher’s feedback, but she maintained her patience by iteratively evaluating the teacher’s feedback and reflecting on the required revisions: “There is a big gap between my essay and the teacher’s feedback. I feel it will be quite difficult to revise based on the teacher’s feedback, but I will try my best to do so.” Her think-aloud data revealed how hard she tried:

The teacher feedback says that there is no argument in this paragraph, and the sentences do not prove my argument. To tell the truth, I feel doubtful about this feedback. I feel that my problem is simply that I lack evidence for my argument. However, I need to revise it. I…

### RQ2: How Do Learners Deploy These Self-Regulated Writing Strategies?

Table 5 shows that cognitive and motivational strategies were used more frequently than metacognitive and social strategies when revising, regardless of the feedback source. The ANOVA confirms significant strategy-use differences according to feedback sources, AWE: *F*(3,105) = 9.331, *p* = 0.000, η^2^ = 0.210; peer: *F*(3,105) = 4.645, *p* < 0.01, η^2^ = 0.117; teacher: *F*(3,105) = 15.354, *p* = 0.000, η^2^ = 0.305.

Table 5 shows that more cognitive strategies were used when revising based on AWE feedback than the other feedback sources, while more motivational strategies were used when revising based on teacher feedback. A repeated measures ANOVA showed that cognitive strategy use was not significantly affected by the three feedback sources, *F*(2,70) = 2.133, *p* > 0.05. However, *post hoc* tests showed a significant difference in cognitive strategy use between AWE and peer feedback sources, *F*(1,35) = 5.096, *p* = 0.03, with a medium effect size (η^2^ = 0.13). Compared with peer feedback (mean = 3.97), learners used significantly more cognitive strategies with AWE feedback (mean = 4.16). Metacognitive strategy use was the highest for teacher feedback (mean = 3.91). However, a repeated measures ANOVA showed that metacognitive strategy use was not significantly affected by the three sources, i.e., *F*(2,70) = 0.475, *p* > 0.05, nor was social strategy use, *F*(2,70) = 0.456, *p* > 0.05. Nevertheless, motivational strategy use was found to be significantly affected by the three sources, *F*(2,70) = 12.178, *p* = 0.000, with a large effect size (η^2^ = 0.26). The *post hoc* tests also showed significant differences in motivational strategy use between AWE and teacher feedback (*p* = 0.003) and between peer and teacher feedback (*p* = 0.000).

Textual analysis of feedback and feedback uptake revealed three feedback quantities for the four participant cases and their feedback uptake rates (Table 6). Notably, AWE feedback is the most frequent overall (72.3–88.5%), although this is mostly at the surface level (over 92.7%), and produces a low feedback uptake rate (11.1–33.3%). Conversely, teacher feedback was less frequent (7–23.1%) but was all aimed at the meaning level (100%) and had the highest feedback uptake rate (100%). Peer feedback was the least frequent (0–16.3%), was mostly at the meaning level, and had the greatest variation in uptake rate (0–55.6%).

**TABLE 6:** Feedback and feedback uptake rate for the four participant cases.

Cases	Feedback sources	Feedback frequency (%)	Feedback type (%)		Feedback uptake overall (%)	Feedback uptake by type (%)	
					
			Surface-level	Meaning-level		Surface-level	Meaning-level
Luan (H)	AWE	72.3	100	0	33.3	17	0
	Peer	4.6	33.3	66.7	4.2	0	50
	Teacher	23.1	0	100	62.5	0	100
Zeng (H)	AWE	88.5	97.8	2.2	33.3	6.7	0
	Peer	0	0	0	0	0	0
	Teacher	11.5	0	100	66.7	0	100
Wu (L)	AWE	76.7	97	3	11.1	3.1	0
	Peer	16.3	28.6	71.4	55.6	100	60
	Teacher	7	0	100	33.3	0	100
Li (L)	AWE	74.5	92.7	7.3	46.7	13.2	66.7
	Peer	14.6	12.5	87.5	13.3	100	14
	Teacher	10.9	0	100	40	0	100

The feedback quantity and uptake of the four cases (Table 6), together with the strategy use of the 36 participants when revising based on three feedback sources (Table 5), revealed an overall tendency of feedback features and the corresponding strategy use for different feedback sources. The high quantity of surface-level AWE feedback seems to demand more cognitive strategies during subsequent revisions, and the high teacher feedback uptake rate at the meaning level appears to elicit more motivational strategy use during revision. Hence, feedback type, quantity, and uptake seem to be associated with self-regulated writing strategy use.

Think-aloud data and interviews indicated high cognitive strategy use in the four cases when revising upon AWE feedback, where they carefully checked each piece of feedback in relation to their original essays and revised accordingly. The high amount of AWE feedback might have led to excessive text processing, which referred to what learners had acquired in both online and offline writing lectures. For example, Li, a low-proficiency student, reported on her decision-making and revision processes in her think-aloud data: “It [Pigai] said ‘basic’ is an adjective but was wrongly used by me as a noun. Yes, it is right. Here, it should be ‘basis’.” Stimulated recall interviews corresponded to the think-aloud data. For example, for the above AWE feedback, Li explained in her interview, “I forgot the part of speech for ‘basic’. I revised it.”

The think-aloud data and interviews indicated high motivational strategy use by the four cases when revising based on teacher feedback. Despite its lower frequency, teacher feedback was all related to meaning, demanding that learners understand the feedback and revise accordingly. The four cases incorporated every item of teacher feedback. Think-aloud data indicated that all meaning-level teacher feedback caused learners to self-reflect and evaluate their original drafts. This process, as the interviews also showed, is characterized by high motivation and constant emotional control.

Notably, learners employed several strategies to achieve their feedback revision goals. The highest and lowest proficiency learners, Luan and Li, are cited here as examples of a series of SRL strategies used when revising based on teacher feedback. Both Luan (high-level) and Li (low-level) employed a series of metacognitive and cognitive strategies when making revising decisions. They both reflected on feedback, evaluated it with reference to their original essays, interpreted the reasons for their inappropriate use, and revised accordingly. They also turned to others for help (social strategies), especially when they were unsure about some indirect teacher feedback. This shows that motivational strategies are vital during this process; they help learners regulate their own motivation to keep revising in a positive frame of mind. Subsequently, they can orchestrate the array of strategies at their disposal to revise effectively.

The teacher said that I should not write a new example at the end of a paragraph, and my example must be included in the essay topic. I had not noticed this before because, when I was writing the essay, I felt this example was similar to another example of the sub-topics. At that time, I thought that I have seen such writing in the reading materials, so I wrote it down. He pointed this out for me, and I revised accordingly (Luan, the highest in proficiency).

The next feedback, to a large extent, is also related to my ineffective expression. I believe that in modern international relations, respect means showing respect to those opposing hegemony and power politics. In this context, I meant the meaning of “respect.” But the teacher said it was very irresponsible to directly point out this viewpoint. He asked me to explain it. Actually, this saying is what I personally believe in, but if it is not explained, it is very difficult to fully convince readers. So I listed some examples here, such as unequal treaties between powerful and less powerful countries, which is what respect means. Thus, adding more specific examples to illustrate the point makes the writing more convincing. (Li, the lowest in proficiency).

## Discussion

This study aimed to examine Chinese EFL learners’ use of self-regulated writing strategies when revising based on AWE, peer, and teacher feedback in an online EFL writing context. Questionnaire data showed that all 36 participants who registered for the online writing course used a relatively high frequency of four self-regulated writing strategies when revising. The multidimensional features of their self-regulated writing strategies are in line with previous studies (Teng and Zhang, 2016).

Motivational strategies were the most frequently used among the four strategy categories when revising based on teacher feedback, which provides support for previous studies acknowledging the role of motivational disposition in self-regulated writing strategies (Vandevelde et al., 2015; Csizér and Tankó, 2017; Teng and Zhang, 2018). Students reported high motivation for handling feedback and revising to improve their writing competence and also controlled their emotions to complete the feedback revision activities. They also reported considerably frequent cognitive strategy use when revising based on AWE feedback. This involved processing and revising texts from linguistic, rhetorical, and discourse aspects with reference to writing knowledge acquired from both online self-study and offline lectures (Sun and Wang, 2020). Metacognitive strategies were also employed, with learners reflecting, evaluating, and interpreting multiple feedback sources with reference to external sources and planning and monitoring revision processes (Bai, 2018; Zhang and Qin, 2018). Social behavior strategies, although the least frequently used, were also reported by participants who resorted to asking others for help with feedback revision activities. The qualitative results of think-aloud data and stimulated recall interviews from the four cases are in accordance with the quantitative findings, demonstrating the use of all four of these self-regulated writing strategies.

These findings echo research into the multidimensional nature of SRL writing strategies (Teng and Zhang, 2016) and learner engagement with feedback (Han, 2017; Zhang and Hyland, 2018). Learners engage in feedback revision based on multiple feedback sources cognitively, behaviorally, and affectively (Dressler et al., 2019) in online writing contexts (Tian and Zhou, 2020). Learners also devote themselves to planning, organizing, analyzing, evaluating, monitoring, socializing, and other self-regulated strategies when engaging with feedback from different sources to complete feedback revision activities (Zhang and Qin, 2018; Xiao and Yang, 2019).

This study also identified the use of different self-regulated revising strategies according to the feedback source. The questionnaire data of the 36 participants showed that they used significantly more cognitive strategies when revising based on AWE feedback and motivational strategies and on teacher feedback. Think-aloud data and stimulated recall interviews coincide with the above quantitative findings. Textual analysis of feedback and feedback uptake in the four case participants indicated an association between self-regulated writing strategy use and feedback features. Learners received the most surface-level feedback from AWE and incorporated revisions based on this feedback source the least. Meanwhile, although learners received less teacher feedback, it was all at the meaning level and resulted in the highest feedback uptake rate.

These results on feedback uptake across multiple feedback sources are in line with previous findings (Han, 2017; Zhang and Hyland, 2018; Tian and Zhou, 2020). Learners’ reported self-regulated strategy use seems to be dependent on feedback type, quantity, and uptake. As surface-level AWE feedback is plentiful, learners require greater cognitive engagement in handling each piece of feedback compared with other feedback sources. Meaning-level teacher feedback has a high uptake rate; seemingly, it is rather demanding to address and make revisions accordingly. Hence, it requires learners to use more motivational strategies to keep themselves motivated and in a positive emotional state (Csizér and Tankó, 2017; Teng, 2021). Therefore, the extent that learners have to devote themselves to different feedback revision processes affects their self-regulated strategy use.

This study’s combination of self-regulated writing strategies and the feedback revision process as a finely focused area of L2 writing responds to Andrade and Evans’s (2013) call for research on SRL strategy use in finely focused areas of L2 writing process.

Seeking, accepting, accommodating, interpreting, and evaluating feedback from multiple sources are vital to fostering self-regulation in such an online writing context (Hattie and Timperley, 2007; Jansen et al., 2017). This study, conducted in an online learning context, has demonstrated that learners systematically activate and sustain cognition, motivation, and behavior to attain feedback revision goals (Zimmerman, 2013), which echoes the role of autonomy in online learning contexts (Murphy, 2005). SRL may then become possible through the regulation of these dimensions in online writing contexts (Xiao and Yang, 2019). Learners in such an online learning environment need to devote more effort to planning, reflecting, monitoring, and evaluating during feedback revision processes (Murphy, 2005). They need to employ an array of cognitive, metacognitive, social, and motivational self-regulated strategies to achieve revising and learning goals.

## Conclusion

This study offers in-depth insights into Chinese EFL learners’ self-regulated writing strategy use when revising based on AWE, peer, and teacher feedback. Both high- and low-proficiency learners actively and systematically used several self-regulated writing strategies to regulate their feedback revision activities.

The implications of this study are three-fold. For learners, verbalizing their thoughts by following think-aloud protocols could raise awareness of their self-regulated writing strategy use and help them self-regulate their feedback revision process. Understanding the self-regulated strategies contextualized in the feedback revision processes could help learners improve the quality of their writing and foster their general self-regulation abilities. For teachers, self-regulated writing strategy training could be implemented, specifically with respect to techniques by which students can orchestrate the array of strategies at their disposal during multiple feedback revision processes rather than identifying one specific strategy. Theoretically, this study’s mixed-methods approach and multiple data collection methods offer L2 writing researchers a way of obtaining comprehensive data to gain insights into the intricacies of finely focused areas of L2 writing.

The small sample size is one limitation of this study. Future research could validate the questionnaire about self-regulated writing strategy use during multiple feedback revision stages through large-scale samples. The variability of learners’ self-regulated writing strategy use is another limitation of this study. Future research could examine more cycles of the feedback-revision process through longitudinal research on different writing tasks to explain how learners can develop self-regulation through feedback revision processes over time.

## Data Availability Statement

The raw data supporting the conclusions of this article will be made available by the authors, without undue reservation.

## Ethics Statement

The studies involving human participants were reviewed and approved by School of Foreign Languages, Renmin University of China. The participants provided their written informed consent to participate in this study.

## Author Contributions

LT conceived of the initial idea, fine-tuned by QL and XZ. LT designed the study, analyzed the data, and drafted the manuscript. QL collected and analyzed the data, revised, proofread, and finalized the manuscript for submission as the corresponding author. XZ collected and analyzed the data, and proofread the manuscript for submission. All authors contributed to the manuscript and approved the submitted version.

## Conflict of Interest

The authors declare that the research was conducted in the absence of any commercial or financial relationships that could be construed as a potential conflict of interest.

## Publisher’s Note

All claims expressed in this article are solely those of the authors and do not necessarily represent those of their affiliated organizations, or those of the publisher, the editors and the reviewers. Any product that may be evaluated in this article, or claim that may be made by its manufacturer, is not guaranteed or endorsed by the publisher.

## Footnotes

1
http://www.pigai.com

## Appendix

**Appendix A:** Self-regulated writing strategy questionnaires when revising upon feedback.

	When receiving feedback from Pigai/peer/teacher and revising, …
**Cognitive strategy**	**Text processing**
	(1) I check for grammar mistakes.
	(2) I check spelling and punctuation.
	(3) I check the structure for logical coherence.
	(4) I check the cohesiveness or connection among sentences.
	(5) I check whether the topic and the content have been clearly expressed.
	(6) I use the English words I know in different ways.
	(7) I try to use different sentence patterns in English.
	**Course memory**
	(1) I revise according to words and expressions taught in online writing lectures.
	(2) I revise according to grammar taught in online writing lectures.
	(3) I revise according to cohesiveness and connection among sentences taught in online writing lectures.
	(4) I revise according to structure for logical coherence taught in online writing lectures.
	(5) I revise according to logical thinking taught in online writing lectures.
	(6) I revise according to words and expressions taught in offline writing lectures.
	(7) I revise according to grammar taught in offline writing lectures.
	(8) I revise according to cohesiveness and connection among sentences taught in offline writing lectures.
	(9) I revise according to structure for logical coherence taught in offline writing lectures.
	(10) I revise according to logical thinking taught in offline writing lectures.
**Metacognitive strategy**	**Idea planning**
	(1) I carefully think about the content of the feedback.
	(2) I evaluate the content to make revising decisions.
	(3) I read related articles to help me revise.
	(4) I categorize the content to be revised.
	(5) I use the internet or dictionaries to search for related information to help me revise.
	**Goal-oriented monitoring and evaluating**
	(1) I set up goals for myself in order to direct my feedback-revision activities.
	(2) I check my revising progress to make sure I achieve my goal.
	(3) I evaluate my revising process.
	(4) I monitor my revising process.
	(5) I tell myself to stick to my revising plan.
	(6) I set up a learning goal to improve my revising.
**Social strategy**	(1) I discuss with others to have more ideas to revise.
	(2) I work with others to revise together.
	(3) When I do not fully understand some feedback, I ask others for ideas.
	(4) When I am not sure about some feedback, I ask others for ideas.
	(5) I share my problems with others so we know what we are struggling with and how to solve our problems.
**Motivational strategy**	(1) I try to improve my English writing based on feedback I receive.
	(2) When I am feeling bored revising, I force myself to pay attention.
	(3) When my mind begins to wander during revising, I make a special effort to keep concentrating.
	(4) When I begin to lose interest for revising, I push myself even further.
	(5) I work hard to revise well even if I don’t like revising.
	(6) Even when revising is dull and uninteresting, I manage to keep working until I finish.
